# HIV Care Seeking Pathways and Barriers to the Continuum of Care Faced by Persons Living With HIV in Rural Nepal: A Qualitative Study

**DOI:** 10.1111/hex.70141

**Published:** 2025-01-05

**Authors:** Amit Timilsina, Anisha Shrestha, Pabitra Neupane, Sudip Nepal, Bishow Kandel, Sudha Devkota, Subash Thapa

**Affiliations:** ^1^ Research and Community Development Center Kathmandu Nepal; ^2^ School of Health Science University of Toledo Ohio USA; ^3^ Department of Gender Studies Tribhuvan University Kathmandu Nepal; ^4^ Universitas Gadjah Mada (UGM) Bulaksumur Yogyakarta Indonesia; ^5^ Central Department of Public Health Tribhuvan University Kathmandu Nepal; ^6^ Government of Nepal Kathmandu Nepal; ^7^ Rural Health Research Institute Charles Sturt University Orange New South Wales Australia

**Keywords:** care pathways, continuum of care, HIV, Nepal, persons living with HIV

## Abstract

**Background:**

The Human Immunodeficiency Virus (HIV) has a major impact on a person's social and personal lives, affecting both physical and mental health. To meet the global 95‐95‐95 target, it is essential to understand and address the multi‐level challenges to improve the continuum of care for persons living with HIV (PLWH). This study delves into the care‐seeking pathways and barriers encountered by PLWH residing in rural areas of Nepal, shedding light on the complexities of accessing and navigating the continuum of care.

**Design:**

This study was designed as a qualitative thematic study that consisted of in‐depth interviews among 21 PLWH and key‐informant interviews among four health service providers in rural districts of Koshi province in Nepal. Semi‐structured interview guidelines were used to ensure consistency in the data collection process, followed by Inductive Coding to identify and categorize the data into codes. Subsequently, sub‐themes and themes were developed, and manifest analysis was conducted to analyze the data. The findings of the study are presented in this paper in the form of excerpts.

**Results:**

The multilevel barriers to HIV care continuum included (i) *socio‐cultural barriers* such as stigma, discrimination, fear of disclosure, and heavy reliance on traditional healers; (ii) *socio‐economic barriers* such as poverty, limited access to health insurance, low health literacy and the exclusion of PLWH under Social Security Act; (iii) *fatalistic lifestyles* characterized by heavy alcohol consumption, and poor adherence to antiretroviral therapy and (iv) *health system‐related barriers* such as mistreatment by healthcare providers, and long distances to ART centers.

**Conclusions:**

There is a need to expand services beyond treatment, including community‐focused awareness and sensitization, programs led by community‐based organization, economic empowerment and inclusion of PLWH under social security mechanisms in rural areas for HIV continuum of care.

**Patient and Public Contribution:**

During the study design phase, two PLWH and two service providers were consulted to discuss the research gap, understand the current practices and discuss the data collection tools and their content. Similarly, four service providers supported implementation of the study and were also consulted to interpret the underlying meaning of the data. One service provider also contributed to the manuscript development process. PLWH and the service providers were also the study participants. The findings of the study are grounded in the data/information provided during the data collection phase, thus meaningfully contributing to this study.

**Abbreviations**
AIDS: acquired immunodeficiency syndrome
ART: anti‐retroviral therapy
CD4: cluster of differentiation 4
HIV: human immunodeficiency virus
PLWH: persons living with human immunodeficiency virus
PrEP: pre‐exposure prophylaxis
WHO: World Health Organization

## 1 Introduction

Human Immunodeficiency Virus (HIV) infection and Acquired Immunodeficiency Syndrome (AIDS), remain a significant global public health concern. Since the onset of the epidemic, it has resulted in a documented death toll of 40.4 million individuals since the epidemic began, while persistent transmission continues to occur across all countries worldwide [1]. HIV infection, coupled with extreme poverty and rural residence can have negative effects on a person's physical health, mental health, social life and overall quality of life including their family members as well as deteriorating the household finances [2, 3]. Access to free treatment and continuity of care is one way to improve this situation. However, extreme poverty, limited healthcare infrastructure and socio‐cultural barriers exacerbate the sufferings of impoverished rural populations diagnosed with chronic infections like HIV, especially in regions with low antiretroviral therapy (ART) retention and enrollment rates [4, 5].

Since the first case of HIV and AIDS, which was reported in Nepal in 1988, in total 71,538 persons have become infected with HIV and 29,890 have died from AIDS‐related illness [6]. Similarly, the factsheet from 2024 estimates 30,300 persons is currently infected by HIV and AIDS and in total 25,728 persons living with HIV (PLWH) are under ART [6]. In Nepal, participation in HIV treatment is predominantly higher among urban men and women. Particularly in the rural areas, PLWH face discrimination and denial of treatment due to their HIV‐positive status, leading to delays in ART enrollment or discontinuation of treatment at some point [4, 5]. Apart from this, various studies conducted in rural areas or in the low‐resource setting have identified several key challenges that contribute to the discontinuation of healthcare services. These challenges include loss to follow‐up, loss to care, low healthcare‐seeking behavior, inadequate facility‐based services, stigmatization, poverty and limited education. These findings suggest the need for population‐specific integrated interventions for continuum of care [5, 7].

Continuum of care refers to the continuous and coherent provision of healthcare services that connect discrete elements in a patient's healthcare journey. It encompasses different episodes of care, interventions by various providers, and improvements in the patient's health status [8]. According to the World Health Organization (WHO), although the initial stage in the continuum of care is ART initiation [9], only 76% of PLWH receive ART, and 71% achieve suppressed viral loads [1]. Previous studies have noted multilevel factors such as socio‐structural, economic and psychological factors that influence treatment continuity among men and women [10, 11, 12, 13]. However, there has been limited focus on rural populations in these studies; as such, the HIV care pathways of PLWH, particularly in rural areas, have not been thoroughly explored.

Therefore, it is crucial to understand the multilevel factors prevalent in specific rural communities to adopt a holistic approach that not only ensures medication availability in rural healthcare facilities but also addresses the unique challenges specific to these rural communities. This study, therefore, is aimed at exploring and understanding the HIV care‐seeking pathways and factors influencing the continuum of care among PLWH in rural Nepal.

## 2 Methods

### 2.1 Study Design and Setting

Qualitative exploratory cross‐sectional study design was employed to examine the perspectives of study participants, highlight similarities and differences, and provide a comprehensive analysis of pertinent issues. The study was conducted at selected ART sites in the Koshi province of Nepal during the months of May to July 2022. Koshi province was selected as a study setting as the ART retention rate among PLWH has been observed to be the lowest (68.9% retention out of total 3260 cases) in the year 2022 compared to any other province in Nepal [14].

### 2.2 Participants' Recruitment and Data Collection

Study participants were individuals aged 18 and above, including males, females and sexual and gender minorities living with HIV from rural areas of selected districts in Koshi province. The respondents were identified through consultations with respective ART counselors and were subsequently approached for their consent to participate in the study while maintaining their privacy. Four health service providers, including two ART counselors and two community public health personnel, were personally contacted, and invited to provide their perspectives and experiences on rural programmatic and policy challenges related to the continuum of care.

Face‐to‐face in‐depth interviews using semi‐structured interview guidelines (Annex 1 and Annex 2) were conducted by hired enumerators with prior experience in qualitative research related to HIV and AIDS. The data collection process involved initial consultations with four PLWHs to better understand the need and current gaps in HIV care, their expectations regarding continuum of care, to pretest the interview guide and interview process. The pre‐tested interviews were not included in the final analysis. All interviews were conducted in Nepali language and subsequently translated into English language for data analysis. Each interview was transcribed with verbatim and then reviewed by the enumerators to ensure data richness and correctness. Data collection continued until data saturation was achieved [15].

In total, 25 participants took part in this study, consisting of 21 PLWH, including 10 from the hilly ART center and 11 from the Terai ART center, along with 4 health service providers/managers (refer Table 1). The ethical approval for the study was taken from the Ethical Review Board of the Nepal Health Research Council before data collection (Reference number: 2769). Written consent was obtained from all participants before data collection. To ensure anonymity of the participants, only the socio‐demographic variables, such as age, gender, ethnicity, marital status, number of children and occupation, were asked. The identity of the participants was pseudonymized during the transcription, translation, coding and manuscript writing to ensure confidentiality and anonymity of the participants and their sensitive information. The interview transcripts have not been shared publicly to maintain the confidentiality of information shared and to maintain the anonymity as agreed with the participants of this study. The study followed the Helsinki Guidelines to adhere with standard research ethics [16].

**Table 1:** Consolidated socio‐demographic table of participants.

S. N	Characteristics		Number	Percentage (%)
1	Age group	20–30	7	28
		31–40	8	32
		41–50	4	16
		51 and above	6	24
2	Gender	Male	16	64
		Female	8	32
		Sexual and gender minorities	1	4
3	Marital status	Married	18	72
		Unmarried	4	16
		Separated	3	12
4	Number of children	No children	9	36
		One and above	16	64
5	Education	No formal Education	5	20
		Primary level Education	5	20
		Secondary level Education	7	28
		Higher Secondary Level Education	3	12
		University level	5	20
6	Occupation	Employed	21	84
		Unemployed	4	16

### 2.3 Data Analysis

Based on the initial review of the transcribed data, latent thematic analysis was carried out to analyze the data as guided by Braun and Clarke [17]. Coding rules for inclusion and exclusion criteria were developed, and inductive coding was used to generate open codes using NVivo 12 software. These codes with similar characteristics and meaningful patterns were then aggregated to form subthemes and themes. We developed themes as broader patterns that connected related codes and sub‐themes, then carefully reviewed and refined them for clarity and uniqueness (Table 2). Finally, we defined and labeled each theme to accurately represent its core meaning, creating a clear, comprehensive, and cohesive narrative that addressed our research question. The findings have been presented in descriptive form under the Results section supported by the relevant excerpts.

**Table 2:** Codebook showcasing the codes, sub‐themes and themes.

Theme	Sub‐theme	Codes
1. HIV Care seeking pathways	1.1. HIV testing and counseling	Awareness regarding HIV; Awareness regarding HIV testing sites; Awareness regarding ART services; Disclosure; Fear of HIV test; Perception on HIV; Identification of HIV status among participants; Prior knowledge of HIV and AIDS; Source of information regarding HIV and HIV care; Stigma regarding HIV and ART; Symptoms before HIV testing and diagnosis; Reason HIV of transmission among participants.
	1.2. Initiation, treatment adherence and retention in HIV care	ART adherence and continuation; Accessibility of ART services; Advice and counselling received; ART experience; Period of ART intake and continuation; Behavior of service providers; Benefit of having ART; Commute distance to and from ART center; Transportation media for ART service; Demotivation and discouragement among participants; Discrimination faced by the participants; Experience of initiating ART; Fear of death; Health‐related behavior of participants; Intervention for vulnerable communities and risk group; Lifestyle of the participants; Mental health of participants; Migration; Motivating factors for ART continuation and adherence; Opportunistic infection status; Personal behavior of participants; Peer influence; Perception on pill burden; Satisfaction from ART services; Side effect of ART; Use of alternative medicine and services; Substance abuse and addiction; Economic situation of PLWH; Suicidal tendency and thoughts; Unemployment; Violence experienced by the participants.
2. Comprehensive HIV care approaches	2.1. HIV literacy and community sensitization	Awareness regarding service availability; Challenges faced by participants in their daily life; Changing perception regarding HIV infected person; Family dependency; Family behavior and attitude; Improved family relation and support; Provision of health insurance; Perception on longevity; Peer support; Perceived quality of life; Need for community centered sensitization program.
	2.2. Integrated community‐based HIV program	Building agency for PLWH; Counselling support for PLWH; Client focused approach in services; Need for community home and center; Evidence‐based program planning; Expectation of participants for improved ART services; Requirement for balanced diet and food; Development of resiliency among participants; Understanding social situation of PLWH; Medical support required by participants; Support by Civil Society Organizations.
	2.3. Economic and livelihood support	Ability to work and earn; Economic status of participants; Financial hardship experienced by participants; Financial support required by the participants; Need for employment or income generation; Need for livelihood support program; Requirement of skill development and vocational education program; Economic support received from the government.

## 3 Results

In total, 73 codes, five sub‐themes, and two themes were developed from the data to present the findings of the study, as stated in Table 2.

### 3.1 HIV Care Seeking Pathways

Most participants became aware of their HIV status only during health checkups prompted by serious illnesses, after which healthcare providers referred them for HIV testing. Following their HIV diagnosis, the majority of participants experienced fear, disbelief and mental health issues, including suicidal tendencies. The majority of the participants were satisfied with the support they received during posttest counseling from healthcare providers and were explained the importance of initiating ART services. ART counselors at governmental hospitals were the first point of contact for all participants to learn about ART and enroll in treatment.

At first, they (service provider) took care of me and explained to me my situation (HIV status). I often talked to them about dying, so he (service provider) advised me not to think that way. He (service provider) convinced me that it is important to take medicines for my health.

(IDI 9)

As soon as the participants received HIV‐positive diagnosis, accessing ART centers and receiving support from ART counselors was an important care‐seeking pathway for them to initiate and continue ART services. One participant with a disability sought support from his family to access ART services.

I am blind. I am a disabled person. I think there should be home‐based services for people like me. I cannot travel alone here to take the medicines, so I have to rely on my children to bring me over here.

(IDI 10)

### 3.2 Comprehensive HIV Care Approaches

The majority of the participants experienced side effects such as headache, stomach pain, weakness, dizziness, fatigue, sleepiness, nausea, vomiting, gastritis, allergies and itches, fever and cough after the they initiated ART services. Some participants discontinued ART services at some point in their life due to experiencing side effects and when their health deteriorated further, they would get back to taking the ART.

I was in India when I first started taking ART. In the beginning, I used to have vomiting, so then I also felt like quitting. I felt like the medicines will make my health worse than it already was. The doctor said that the side effects would go away eventually. So, I continued taking the medicines and the side effects started to disappear over time. Now I experience no such side effects.

(IDI 7)

The majority of the participants felt positive about continuing ART. The benefits of ART enrollment included improvements in their health and underlying conditions, relief from pain, gastritis, and dizziness, weight gain, prevention of opportunistic infections or relapse of wounds and blisters, increased appetite and decreased viral load. The participants described ART as helping them pursue a ‘normal’ life, fulfill their dreams, and prolong their lives.

If I had not taken the medicine, I would have been at risk of being infected with other diseases as well. I might have got many kinds of diseases. I would have got a disease that I do not currently have and, I might have faced mental health problems. This medicine has helped me a lot.

(IDI 5)

a. **Socio‐cultural Barriers to Continuity of Care**

Participants explained familial and societal misconceptions towards PLWH and being abandoned by partners and family members. Consequently, the fear of disclosing their HIV status has been seen as an important factor in discontinuing the treatment. Some participants, particularly those with disabilities and from sexual and gender minority communities, have also experienced violence and abuse. Similarly, one of the reported coping strategies was taking the medicines in private but not in front of the family members. Some participants did not disclose to the family members and friends about receiving ART services.

My wife is abroad, and I have not seen her in 15 to 20 years. She might have known about my HIV status, which could be the reason why she did not want to see me. I have had phone calls 3 or 4 times during these 15 to 20 years. We do not live together.

(IDI 16)

b. **Economic Barriers to Continuity of Care**

For the most participants, the ART center was far from home, and out‐of‐pocket expenditure including costs for transportation, accommodation, meals and hospitalizations associated with ART treatment were hard to manage. The participants with low‐income levels had hard time making decisions about even visiting the health centers. All study participants were either unaware of or had no access to the National Health Insurance Program to cover certain levels of medical expenses. To cope with the financial problems, some participants had to take loans from friends and relatives to receive treatment when they were sick and to seek ART services, even though HIV treatment is free in Nepal.

I find it difficult when I get sick. I have to borrow money from other people if I get sick. But sometimes I cannot even find the money to come to the hospital.

(IDI 8)

The majority of the participants highlighted the importance of economic empowerment and creating job and livelihood opportunities for PLWH. Those who were unemployed emphasized the need for providing vocational education and skills, along with financial support to start livelihoods and businesses. They suggested that the federal, provincial and local governments should also prioritize and allocate quotas for PLWH in both government services and the private sector to help support their livelihood.

The main issue is money. We cannot do anything without it. We need money for everything. If I had money, I would start goat farming or pig farming. But what can I do? I have no money. Currently, I am engaged in agriculture by leasing land from others on a sharecropping basis (adiya). Fortunately, I have support from my father and elder brother.

(IDI 8)

Other alternative support as suggested by the service providers was an inclusion of PLWH under social security schemes, such as provision of allowance, extended health insurance package by federal, provincial, or local government to address the financial hardship.

If patients with chronic diseases receive allowances from the government, why can't Person living with HIV receive such allowances to support their daily life necessities? We have been advocating for an allowance to cover out‐of‐pocket expenditures for Person living with HIV and AIDS, but it has not been successful.

(KII 3)

c. **Poor HIV Literacy and Unhealthy Lifestyle**

The participants who discontinued ART services reported limited knowledge about HIV, long‐term stress, carelessness and fatalistic lifestyle, including substance abuse, unemployment, neglect of family responsibilities and unsafe sexual behaviors. The participants highlighted the need for increased awareness and community sensitization regarding healthy lifestyle choices, and the importance of love, care, and support for a healthy and happy life.

I felt hopeless and started consuming alcohol. During that time, I even stopped taking ART. I thought my life was coming to an end.

(IDI 20)

d. **Health System‐related Barriers to Continuity of Care**

Participants had mixed experiences regarding the attitude and behaviors of service providers, which significantly impacted their treatment adherence. Some participants also reported instances of service providers being rude, insensitive in their questioning, and exhibiting discrimination and prejudice, leading to discontinuation of ART services or demotivation among the participants. However, the majority of participants who continued with the services highlighted positive aspects such as counseling, friendly behavior, follow‐up, and ART medication delivered to their door. For instance:

At times, I couldn't go to the ART centers monthly, so the service providers used to provide medicine to cover 2 months, which helped me continue my medication.

(IDI 16)

The long distance to ART centers was also associated with practical difficulties such as traveling during rainy seasons, keeping track of time and managing time from work. Despite these challenges, the improved health and the fear of death if medicine was discontinued were reasons for their continuation with treatment. Few participants reported the pill burden of ART and were having a difficult time continuing the treatment. One of the participants also suggested scientists to develop vaccine or one‐time oral medication.

Yes, the burden is there. I have to carry my medicine with me because I work in the field (agriculture). I go to the field in the morning and return directly in the evening. There's a risk of the medicine dropping or getting lost. Sometimes I forget to take it, which makes me feel burdened.

(IDI 14)

Service providers' continued support through counseling, initiating ART services, ensuring treatment adherence, conducting follow‐ups, and exhibiting friendly behavior has been an important factor in the continuum of care. Additionally, participants have highlighted the importance of having medicine availability as needed and decentralizing ART centers at the local level to reduce the hardships related to distance, economic challenges and opportunity costs associated with their daily lives. Participants who received support from nongovernmental organizations emphasized the role of these organizations in dispensing medicine at the local level. One PLHIV with disability shares:

I am a disabled person, so I believe there should be services for me. I cannot come alone to the clinic to get my medicine, so my children have to bring me here. If I could receive home delivery of medicines, I could get my medicine at home and my children's studies would not be interrupted.

(IDI 10)

Some participants, including key‐informants, feel the need to expand services beyond treatment, which includes periodic examination of viral load and CD4 cell count, availability of nutritious food, access to self‐care practices and mental health counselling.

There should be a provision of viral load examination on a periodic basis, examination of CD4 cell count, and balanced nutritional diet services should be added.

(IDI 16)

## 4 Discussion

This study explored HIV care seeking pathways and barriers to the continuum of care for PLWH in the rural areas of Nepal. Most participants became aware of their HIV status only after experiencing severe illness and undergoing a health checkup. ART counsellors at the governmental hospitals were the first point of contact for all participants where they learned about ART and enrolled in the treatment. Majority of the participants expressed satisfaction with the support they received from the healthcare providers during ART enrollment. This study highlights importance of navigating these pathways and maintaining continuum of care was challenging due to multilevel challenges in the rural areas. These challenges included socio‐cultural barriers, economic barriers, poor health literacy and fatalistic lifestyles, and health system‐related barriers.

The lack of awareness regarding preventive measures and inadequate access to prevention and treatment services is concerning that a widening rural HIV treatment and prevention gap are the major reasons for risky behaviors, late diagnosis and low ART retention in the rural areas suggested by this study. Moreover, this study also pinpoints the limited coverage of national health insurance, low number of community and home‐based care as well as dispenser sites for vulnerable and at‐risk populations, and low number of free testing services for PLWH as major barriers in HIV continuum of care, despite substantial investments in scaling up HIV treatment and prevention in Nepal [2].

Multiple studies across the globe have identified the community and home‐based care model to be an effective approach in delivering healthcare and these studies highlight that support, training and reasonable renumeration as motivating factors for efficient operation of community and home‐based care [18, 19, 20, 21]. Apart from the community and home‐based care, the multi‐level barriers indicate an urgent need to scale up of targeted community‐based awareness, intervention and harm reduction strategies in rural Nepal. Despite the efforts from the Government of Nepal, alarmingly low rates of testing and ART enrollment indicate a significant risk of HIV transmission from high‐risk populations to low‐risk general populations [6, 22].

Upon receiving their HIV‐positive diagnoses, this study highlights that individuals generally experience psycho‐social and emotional problems, including anxiety, chronic stress and suicidal thought. These problems can be addressed through psychological support, but these needs have been largely neglected in rural areas. Previous studies conducted in Nepal are consistent to findings of this study suggesting poor mental health status among people living with HIV and AIDS [22, 23, 24].

This study recommends quality client‐centric ART services, decentralized health centers and comprehensive information and education regarding HIV. It also emphasizes on the importance of constant motivation, adequate counseling and follow‐up by healthcare providers, as well as encouraging the clients to have self‐belief regarding positive health outcomes of ART, which can lead to treatment adherence and continuation of ART services [25]. The findings of this study are also consistent with the study conducted by Shrestha et al., which has suggested a comprehensive approach beyond treatment which includes improving the attitude of service providers, bolstering emotional and social support and family counseling [12]. Our study also supports and fosters the important and catalytic role of healthcare professionals, coupled with effective counseling and follow‐ups which have contributed to ART treatment initiation, retention and adherence.

Side effects of ART medications, underlying mental health issues, social stigma, long distance to the nearest ART centers, and a lack of support within family compounds the problems PLWH face while continuing ART services [11, 25]. The problems include financial constraints, perceived stigma and prejudice, medicine side effects, clinic‐based ART provision, including long waiting times, poor confidentiality, restricted opening hours and dissatisfaction with healthcare services have been also revealed by studies conducted in African countries [3, 26].

A recurring theme in this study is the need for economic and financial support and empowerment for financial viability are crucial, particularly for PLWH with intersectional identities, which includes PLWH from rural areas, with disabilities, and sexual and gender minority communities, which this study focuses on. Reducing the financial burden on HIV‐affected persons and households appears to be possible through decentralized treatment services in each district and offering social support and income‐generating programs to affected individuals and their families [27]. The global funding on HIV and AIDS has been a challenge over the years and the findings of this study suggest there are still substantial health and social needs that remain unmet and unaddressed, especially in the resources‐limited settings such as Nepal. To complement the gap, the findings of this study stresses, the pivotal role Provincial and Local Government can play in empowering PLWH through livelihood programs, skill development for employment, and affirmative action to create job opportunities for PLWH in both the public and private sectors.

The plethora of evidence suggests significant potential the micro‐finances hold to reduce poverty by providing livelihood opportunities and decreasing household poverty [28, 29, 30, 31]. Another crucial intervention this study suggests to ensure the HIV continuum of care is the inclusion of PLWH under the social security schemes of Nepal and expanding the range of service support, such as regular assessments of viral load and CD4 cell count, and financial support for PLWH through health insurance coverage. HIV‐related illnesses are still disproportionately excluded out of insurance plans offered by the public and private sectors, and inclusion of such programs in the insurance coverage can improve the health security of migrant and mobile workers are subsequently improved by such programs [32]. Public–Private partnership modalities have been proven to be an effective way to improve PLWH insurance coverage [32]. One of the novel findings of this study is the reluctance of the policymaker to include PLWH under the provision of allowances. For instance, patients with cancer, renal disease and spinal cord injury disease are provided with Rs. 5000 as a living allowance to cover nutritious food, medical expense and transportation, but the PLWHs are not included under the critical illness and thus not included under provision of allowance [33].

Meeting the global 95‐95‐95 target by 2030 seems less optimistic given the challenges of ART enrollment, treatment adherence and continuity of care faced by rural populations and if the subnational, national and global actors do not invest to strengthen the care seeking pathways and continuum to care. There is a necessity of integrated and comprehensive approach and mechanism for a continuum of care including adequate monitoring that circumvents case identification and management to achieve the goal of ending the AIDS epidemic as a public health problem, meeting the 95‐95‐95 target, and prioritizing the quality of life of PLWH [34, 35].

A systematic self‐care mechanism could be an alternative care pathway for PLWH and promote help‐seeking behavior in the community. Client‐driven approaches, such as HIV self‐care interventions which include the awareness and availability of self‐testing, self‐medication (such as PrEP and Opioid Substitution Therapy), online counseling, online dispatch of ART medicine, and telemedicine service can help bridge the geographical challenge and commute hardship. There has been significant progress in HIV testing and counseling for overall HIV treatment, care, and support services in Nepal. HIV self‐testing is scaled up across key population in Nepal and the National HIV Strategic Plan 2021–2026 also encourages the implementation of cutting‐edge strategies like PrEP, online‐to‐offline testing, expanded peer outreach, index testing, HIV self‐testing, and new technologies for virtual support and follow‐up mechanisms. These initiatives aim to strengthen the HIV care and support services in Nepal.

### 4.1 Strengths and Limitations of This Study

Given the purely exploratory nature of this study, the qualitative method employed has produced substantial insights into the phenomenon studied, serving as a valuable groundwork for future research and interventions in this understudied research area within the field of HIV. The study has helped to bridge the existing knowledge gap in the understudied area of existing HIV care seeking pathways and HIV continuum of care which helps policymakers, health managers and HIV health cadres to better understand the priorities to end HIV and AIDS. The findings of this study can be instrumental for other local and middle‐income countries, development agencies and actors for strategic discussions and interventions aimed at meeting the global 95‐95‐95 target.

However, due to a highly selective sample, our findings may not have captured the interests and experiences of a comparatively broader population and all contextual insights. Additionally, the question guide used in this study was neither validated nor pilot tested. The socioeconomic context, HIV care context, and needs of PLWH from other provinces of Nepal may differ. Hence, a national‐level, large‐scale study is required to obtain generalizable evidence. Similarly, due to specific scope of this study, the perspective of policymakers and actors at the subnational and national levels may not have been explored fully in this study to design, integrate and implement community‐based interventions and resources for strengthened care pathways and continuum of care.

## 5 Conclusion

This study emphasizes the barriers to the continuum of HIV care in rural areas, where challenges include stigma, economic hardships, low health literacy, and limited access to healthcare. Factors such as poverty, lack of health insurance, reliance on traditional healers, and stigma hinder ART adherence among people living with HIV. Health system issues including mistreatment by healthcare providers and a lack of community‐based care, further complicate continuation of ART services among PLWH. To address these issues, we advocate for comprehensive community‐based strategies, particularly in resource‐poor settings like Nepal. Key recommendations for HIV continuum of care include expanding HIV services (self‐care, PrEP, OST, viral load tests), promoting the availability of ART medications, cross‐border collaboration, and the inclusion of PLWH under social protection programs. Additionally, community sensitization, family and community support, and economic empowerment through job‐oriented training and job creation are essential. The call for safety nets for continuum of care for PLWH, which this study highlights, could provide strategic direction and support to the Government of Nepal, development agencies and stakeholders, to guide strategic discussions and intervention for comprehensive and tailored strategies to improve ART adherence and ensure HIV continuum of care among PLWH, aiming to meet the global 95‐95‐95 target and ending the AIDS epidemic by 2030.

## Author Contributions

**Amit Timilsina:** conceptualization, data curation, formal analysis, funding acquisition, investigation, methodology, project administration, software, validation, data curation, writing–original draft, writing–review and editing. **Anisha Shrestha:** conceptualization, data curation, formal analysis, funding acquisition, investigation, methodology, project administration, writing–original draft, writing–review and editing. **Pabitra Neupane:** writing–review and editing. **Sudip Nepal:** writing–original draft, writing–review and editing. **Bishow Kandel:** writing–review and editing. **Sudha Devkota:** conceptualization, writing–review and editing. **Subash Thapa:** conceptualization, supervision, writing–review and editing.

## Conflicts of Interest

The authors declare no conflicts of interest.

## Data Availability

The data that support the findings of this study are available on request from the corresponding author. The data are not publicly available due to privacy or ethical restrictions.

## Annex 1 Semi‐Structured Interview Guideline for In‐Depth Interview

**Date:**

**IDI number:**

**Place:**

**Start time of interview: End time of interview:**

Socio‐demographic determinants:

1. Please tell me about yourself (Probe: age, sex, education, marital status, occupation).
2. When did you start ART service? What motivated you to join ART service? (Probe: How did you know about ART service? Did you have appropriate knowledge and information regarding ART service before initiating?).
  Structural determinants:
3. How has your ART experience been?
4. What motivated you to take ART service continuously?
5. Did you fear initiating ART service? Why? (Probe: How did you deal with it?).
6. Does the ART service help you or not? How?
7. Are you satisfied with the quality of service provided? Why, why not?
8. How do healthcare providers treat you? (Probe for service provider biasness, harassment, abuse, support, follow‐up, motivation).
9. What motivates you to continue or discontinue your ART service or return to the treatment? (Probe: How did it affect you to discontinue ART service or continue ART services?) (Note: Dig in more for specific underlying causes the participant mentions about. Example may include but not limited to travel distance, financial reasons, stigma and discrimination, service provider bias, migration and perceived benefit/harm of ART).
  Social determinants:
10. What do you do for a living? Do you have any other economic sources? Is it enough for you to live a healthy life? Why, why not?
11. What is societal perception towards your HIV status? Do you feel any sort of discrimination? How do you deal with it?
12. Have you disclosed your HIV status to a family member or community? Why or why not?
13. Have you experienced any sort of harassment or violence from community people?
14. Can you please explain your daily routine from morning until you sleep? (Probe: housing, basic amenities and the environment, dietary pattern, gender roles).
15. How is your dietary pattern or what do you have for breakfast, lunch, dinner? (Probe for balanced diet).
16. Any alternative healthcare treatment that you have sought? (Probe: Dhami Jhakri, mata, jabibuti).
  Behavioral determinants:
17. Do you take any substance such as alcohol, smoking or drugs?
18. How do you define your relationship with your family, friends and community people? (Probe for relationship turbulence).
19. Do you have multiple sexual partners now? Do you have unprotected sex?
20. Do you think you can lead a healthy life? Do you think ART can help you lead a healthy life? What do you think can help lead a healthy life?
21. Do you feel a burden with ART treatment (pill burden)? How do you deal with it?
22. How do you want to make your life productive in the coming days and years?

We are at the end of our interview. Do you want to ask anything or share anything that has not been asked in this research?

Thank you for your participation in this interview.

## Annex 2 Semi‐Structured Interview Guideline for Key Informant Interview

**Date:**

**KII number:**

**Place:**

**Start time of interview: End time of interview:**

Socio‐demographic determinants:

1. Please tell me about yourself (ethnicity, age, gender, occupation, years of experience).

Structural determinants:

1. What are the existing facilitators and barriers in retaining ART client?
2. Are the people living with HIV satisfied with the current ART services?
3. What can be done to improve structural barriers to continue ART services and ensure continuum of care among people living with HIV?
4. What can be done to reduce the impact of perceived pill burden among people living with HIV?
5. What are the existing policy and systemic barriers that hinder continuum of care among person living with HIV? What can be done for progressive policy and practices to foster a continuum of care?

Behavioral determinants:

1. How can the harmful behavior and practices among the person living with HIV be reduced for quality of life?
2. What could be your role in supporting a person living with HIV for positive behavior?
3. How can the fear of disclosure and fear of death be reduced?
4. How can the distrust towards the ART be reduced?
5. How do you motivate a person living with HIV to promote the continuation of ART services?

Social determinants:

1. The participants mentioned stigma, social discrimination and hatred after disclosure of HIV status. What can be done to aware community people regarding the HIV and AIDS, importance of support towards persons living with HIV for continuum of care?
2. What could be community‐based interventions and programs to help the continuity of HIV care and services?

We are at the end of our interview. Do you want to ask anything or share anything that has not been asked in this research?

Thank you for your participation in this interview.
